# Design and Feasibility of a Randomized Controlled Pilot Trial to Reduce Exposure and Cognitive Risk Associated With Advanced Glycation End Products in Older Adults With Type 2 Diabetes

**DOI:** 10.3389/fnut.2021.614149

**Published:** 2021-02-15

**Authors:** Roni Lotan, Ithamar Ganmore, Abigail Livny, Shahar Shelly, Moran Zacharia, Jaime Uribarri, Paul Beisswenger, Weijing Cai, Michal Schnaider Beeri, Aron M. Troen

**Affiliations:** ^1^The Nutrition and Brain Health Laboratory, The Robert H. Smith Faculty of Agriculture Food and the Environment, The Institute of Biochemistry, Food and Nutrition Science, The Hebrew University of Jerusalem, Rehovot, Israel; ^2^The Joseph Sagol Neuroscience Center, Sheba Medical Center, Tel HaShomer, Israel; ^3^Memory Clinic, Sheba Medical Center, Tel HaShomer, Israel; ^4^Neurology Department, Sheba Medical Center, Tel HaShomer, Israel; ^5^Sackler Faculty of Medicine, Tel Aviv University, Tel Aviv, Israel; ^6^Division of Diagnostic Imaging, Sheba Medical Center, Tel HaShomer, Israel; ^7^Department of Neurology, Mayo Clinic, Rochester, MN, United States; ^8^Icahn School of Medicine at Mount Sinai, New York, NY, United States; ^9^PreventAGE Healthcare, Lebanon, NH, United States

**Keywords:** mild cognitive impairment, type 2 diabetes, diet, randomized controlled trial, pilot, feasibility, advanced glycation end products

## Abstract

**Introduction:** Advanced glycation end products (AGEs) in diet and serum are positively correlated with chronic conditions such as type 2 diabetes and cognitive decline. Dietary reduction of AGEs was shown to reduce their level in serum and to have a beneficial effect on metabolic biomarkers. However, in part due to limitations of feasibility, clinical trials have not tested its effect on cognition in elderly. The current pilot study examines the feasibility of AGE reduction in elderly with diabetes in terms of recruitment and retention.

**Methods:** The design is a randomized controlled pilot trial of dietary AGEs in elderly with type 2 diabetes (clinicaltrials.gov NCT02739971). Recruitment followed two stages: we first recruited participants with mild cognitive impairment (MCI), and after expanding inclusion criteria, we later recruited cognitively normal participants with subjective memory complaints (SMCs). Participants were randomized to two arms. Participants in the control arm received standard of care (SOC) guidelines for good glycemic control; those in the experimental arm, in addition to SOC guidelines, were instructed to lower their dietary AGE intake, primarily by changing their cooking methods. Participants were closely followed for dietary adherence over 6 months and evaluated before and after the intervention for adherence to the assigned diet, blood tests, cognitive performance, and brain MRI.

**Results:** Seventy-five participants (52 with MCI and 23 cognitively normal with SMCs) were recruited primarily through mass mailing and advertising in social media websites. Seventy participants finished the study, and dropout was similar in both groups (7.5% in control vs. 5.7% in intervention, *p* = 0.757). The majority (57.5%) of participants in the AGEs-lowering arm showed very high adherence with the dietary guidelines.

**Discussion:** Targeting feasible lifestyle modifications in high-risk populations could prevent substantial cases of cognitive decline. Observational evidence supports that AGEs may contribute to cognitive decline; however, the cognitive effect of reducing AGEs exposure has yet to be evaluated in a randomized controlled trial (RCT). The results of our pilot trial delineate a methodology including effective recruitment strategies, population of choice, and ways to assure high adherence during lifestyle modifications, and significantly advance progress toward a definitive and well-powered future RCT.

## Introduction

The elderly are the fastest-growing segment of the Western population (1), and cognitive decline affects increasing numbers among them. Dementia, the clinical manifestation of severe progressive brain disease and cognitive decline, is projected to triple by 2050 and affect above 115 million people worldwide (2). Although Alzheimer's disease (AD) is the most common cause of dementia, vascular pathology, inflammation, and oxidative stress are prominent features of almost all types of dementia (3).

The failure of numerous clinical trials with candidate drugs to treat dementia has refocused attention on the potential of lifestyle interventions to delay or prevent the disease, including improvement of physical activity and diet, and management of risk factors for chronic cardiometabolic diseases like type 2 diabetes (T2D) (3). Given the consistent association of T2D with increased risk for dementia and AD (4, 5), vascular dementia (6), mild cognitive impairment (MCI) (7), and rapid cognitive decline (8), cognitive impairment and dementia are increasingly considered to be a serious complication of T2D in older adults (9).

Increased exposure to circulating advanced glycation end products (AGEs) in both T2D and AD offers a mechanism to explain these associations and suggests that lifestyle intervention could lower risk through reducing intake of AGEs (10). AGEs is a class of chemical compounds generated endogenously in the course of normal metabolism by the non-enzymatic reaction between reducing sugars or dicarbonyls and free amino groups of amino acids such as lysine and arginine. AGEs tend to accumulate with aging (11) and under conditions where the precursors for the reaction, such as glucose, are in excess. Thus, elderly individuals with T2D tend to have higher levels of circulating AGEs (12, 13). In addition to the endogenous creation of AGEs, exogenous sources such as pre-formed AGEs from food, particularly from animal-derived foods, also contribute significantly to the body's pool of AGEs. Cooking and exposure to high temperatures (i.e., grilling, broiling, roasting, searing, and frying) and long cooking times can greatly increase the amount of AGEs in food (14, 15).

Elevated circulating AGEs are correlated with a wide range of chronic diseases includingT2D and its complications (11, 16). Several clinical trials have demonstrated that by changing their cooking methods, people can reduce their circulating AGE levels and improve glucose metabolism, insulin resistance, and biomarkers of inflammation and oxidative stress (17–19). Growing evidence also suggests an association of AGEs with neurodegenerative disorders including AD (20). This association was shown in observational studies in humans (21) and in animal models (22). In older adults, higher levels of dietary or serum AGEs are associated with faster cognitive decline over time (21, 23). Evidence from animal studies showing that mice fed high-AGEs diet had lower performance scores on cognitive tests and higher hippocampal levels of insoluble amyloid also support a direct involvement of AGEs in the brain (24). Small post-mortem studies of AD brains found AGEs in neurons, astrocytes, and glial cells (25–28) as well as colocalization with the pathological hallmarks of AD, namely, amyloid plaques and neurofibrillary tangles (27). Recently, in humans, serum AGEs levels have been shown to be highly correlated with brain levels of AGEs (29). In addition to direct effects of AGEs on the brain and blood–brain barrier (30, 31), AGEs may also drive systemic inflammation and oxidative stress that play an important role in AD.

Given this biological plausibility and the results of clinical trials that have demonstrated that AGEs in serum are sensitive to dietary AGEs reduction, AGEs are likely a modifiable risk factor for cognitive decline.

We recently conducted a pilot randomized controlled trial (RCT) to develop methodology and evaluate the feasibility of intervening to lower dietary AGEs and of tracking the effect on cognitive performance and brain-related outcomes in older adults with T2D. The biological response to reducing dietary AGEs intake on circulating AGEs and other metabolic biomarkers is discussed in detail elsewhere (32).

Here, we focus on a detailed description and analysis of the study methods, including the dietary intervention, study participant selection, recruitment, and adherence strategies, so that these may be applied to future trials.

## Methods

The study was designed according to the CONSORT extension criteria for pilot trials (33) and was approved by the Helsinki Committee of the Sheba Medical Center, Israel. All participants signed informed consent. The study was registered with www.clinicaltrials.gov (NCT02739971).

### Participant Recruitment and Eligibility

This pilot initially aimed to study elderly participants with T2D and with MCI (stage 1). As the rate of recruitment was lower than expected, we included participants with subjective memory complaints (SMCs) who did not meet the criteria for MCI according to our screening tools, and they were classified with normal cognitive performance (stage 2). Recruitment strategies included:

1. Mass mailing to a list of 30,000 people who asked to be informed on health issues through the company “Infinity”;
2. Publication of the study in websites and forums for clinical trials, dementia, and diabetes;
3. Lectures at nursing homes on brain health;
4. Referral of participants with T2D from ongoing observational studies/screen failure from other RCTs at the Joseph Sagol Neuroscience Center at Sheba Medical Center;
5. Presenting the study to the health care staff at a diabetes clinic in “Maccabi,” a health medical organization with a request that they refer suitable candidates;
6. Publication of the study in the local newspapers, in the area of the hospital, with distribution of 30,000 units.

Interested people left their contact details through a voice machine or on a designated electronic landing page where they could answer questions related to inclusion criteria such as age and diagnosis of T2D.

Suitable candidates were contacted by phone to ascertain that eligibility criteria were met [e.g., age, diabetic subject, not suffering from dementia, have informant (a family member with a constant relationship with the candidate), not vegetarian (as vegetarians eat very low amounts of AGEs), and have SMCs].

### Face-to-Face Screening

Inclusion criteria for type 2 diabetic adults aged 65 years and older are summarized in Table 1. Eligible participants were asked to bring medical records to the screening meeting. Participants whose records indicated T2D, those taking medications for T2D, and those whose last two blood tests for fasting glucose were above 126 were considered as having T2D. Additional screening tools were used to ascertain cognition and dietary AGEs intake. The following screening tools were used:

- Clinical Dementia Rating (35) scale score of <1 (CDR = 0.5 at the first stage of study recruitment and CDR = 0 after normal controls became eligible),
- Montreal Cognitive Assessment (MoCA) (36) score of >18 (score between 18 and 26 in the first stage of the study recruitment and above 18 after normal controls became eligible),
- Geriatric Depression Scale (37) score of <6, confirming lack of mild or major depression (same score in both stages).

**Table 1:** Eligibility criteria.

➢ Above the age 65
➢ T2D diagnosis
➢ No dementia (i.e., MCI or cognitively normal)
➢ Not receiving cholinesterase inhibitors
➢ No other neurological (e.g., stroke, Parkinson's disease) or psychiatric conditions (e.g., schizophrenia, depression) that may affect cognition
➢ Dietary AGE levels ≥ 13 kU
➢ Not vegetarian
➢ Not participating in another clinical trial
➢ An informant that is willing to actively support the participant throughout the study
➢ Not suffering from chronic kidney disease^a^

a *Chronic kidney disease was calculated by the Cockcroft and Gault equation (34) based on the subject's medical record of creatinine in the last 6 months. Participants with creatinine clearance >60 ml/min was considered eligible*.

### Dietary AGEs Questionnaire

To assess AGEs' consumption, we used a Hebrew version of a validated 7-day AGEs-specific food frequency questionnaire with food items rich in AGEs. The English version of the AGEs questionnaire is attached in Supplement A. The information on participants' eating habits included he frequency at which such foods as meat, fish, poultry, cheese, egg yolk, fats, fast foods, and convenience breakfast and snack foods were consumed in the last 7 days; the portion sizes; and methods of cooking (boiling, roasting, broiling/grilling, frying, or canned). Each item is assigned a numerical value, multiplied by the number of entries; the sum of entries per day provides an AGEs score. The AGEs score from this questionnaire has been significantly correlated with AGEs data derived from a 3-day record and with serum AGEs (38). The AGEs questionnaire was developed based on a database of ~560 foods that list AGEs values expressed as AGEs Equivalents (Eq/day), AGEs equivalent = 1,000 kilounits (kU) (15). Participants with score above 13 kU, the cutoff used in other clinical trials for AGEs reduction, were eligible (39).

### Dietary Guidelines

Participants enrolled in the study were randomly assigned to one of two diets by a computerized random sequence generator. Participants were asked to maintain the instruction for the study for 6 months and to avoid any new diet or lifestyle program during this period. At baseline, participants from both groups were guided face to face, by a registered dietitian, to follow guidelines to maintain good glycemic control (40). The main focus of the guidelines was on intake of carbohydrates including information on serving sizes, counting, and quality. Recommendations were given for carbohydrate intake from vegetables, fruits, whole grains, legumes, and low-fat dairy products while reducing the consumption of refined sugars. In addition, participants were advised to prefer food items rich in mono- and polyunsaturated fatty acids compared to saturated and trans fatty acid and to consume lean meat, low sodium, and low processed food items. Other guidelines for good glycemic control were individualized and were based on the participants' nutritional and metabolic needs, habits, preferences, and willingness and ability to make behavioral changes. Those assigned to the AGEs-lowering group were guided, in addition, on how to reduce their AGEs in diet mainly by modifying the cooking methods and lowering cooking time and temperature, without changing the quantity or nutrient composition of their eating habits. They were advised to boil, poach, stew, or steam and to avoid frying, baking, or grilling of animal-derived products (15). The participants received examples for dishes with relatively low AGEs content based on their eating habits (e.g., a subject who reported eating fried meatballs was advised to put the meatballs in tomato sauce without frying them first, and a subject who reported eating roast chicken was advised to make chicken soup, etc.) (15). All participants received written guidelines. Informants who shared a house and cooking with the subject were asked to join the nutritional counseling. If they could not attend, the dietitian instructed them *via* phone.

Participants from both groups were monitored on a weekly basis by telephone conversation to ascertain and encourage adherence with their diet. In the AGEs-lowering group, feedback was specifically given to maintain low AGEs intake. Participants were asked whether and how many times they cooked with the cooking methods causing higher AGEs since the last call. If a participant reported consuming food items rich in AGEs, the instructions to lower AGEs was repeated to both the subject and the informant. Participants from both groups were asked general questions about their adherence to the good glycemic control guidelines (e.g., “did you manage to follow the guidelines for your diabetes?” “did you have any occasions in which you did not manage to eat according to the guidelines”). The telephone conversation content was summarized by the study dietitian including the date of the conversation and main issues raised. Each following conversation included general questions to all participants as well as tailored questions and comments related to the prior conversations on difficulties that were raised (such as preventing hypoglycemia, handling holidays, and special events) and progress (such as self-monitoring of fasting glucose) of the specific participant. The telephone conversations lasted between 5 and 15 min, depending on how much was needed to re-discuss the recommendations for those who did not comply with their respective diet.

### Measuring Adherence to Diet

Diet adherence was categorized by the study dietitian at the end of the study and before the results of AGEs in serum or any analysis of outcomes to avoid bias. Following each phone conversation with the participants, the dietitian elicited answers to two questions: (1) Since our last call, did the participant use any cooking methods they were advised to avoid for certain food items? (yes/no for low AGEs only), (2) Since our last call, did the participant completely maintain the guidelines for good glycemic control? (yes/no for all participants). As carbohydrates intake is the key strategy in achieving glycemic control (40), the answer for maintaining guidelines for good glycemic control was focused on the intake of food items from this group.

Participants in the AGEs-lowering arm were scored twice; once for their adherence to the AGEs reduction and once for their adherence for good glycemic control. Participants in the standard of care (SOC) arm were scored only for their adherence with the guidelines for good glycemic control. Phone calls with “yes” answers (reflecting full compliance with guidelines for T2D or AGEs reduction) were granted a score of 1. If participants reported they had not followed instructions for good glycemic control (focused on carbohydrates) or had used cooking methods that promote AGEs creation in animal-derived products (applied for AGEs-lowering arm) *even once* since the last call, the score of the conversation was 0. The scores for phone conversation (0/1) were then summed for each subject and were divided by the average number of phone conversations. For example, if the average number of phone conversation was 17, and the subject reported in 12 conversations that they had faithfully maintained the dietary instructions, then adherence was scored as 12/17^*^100 = 70%.

Table 2 displays the characteristics for each score for both arms. Adherence was measured on a 1–5 Likert scale based on the participants' report if they maintained AGEs or T2D recommendations: 1 for very high adherence, 2 for good adherence, 3 for partial adherence, 4 for lack of adherence but intention to adhere more in the future, and 5 for lack of adherence and no intention to adhere more (e.g., desire to withdraw).

**Table 2:** Adherence scale with dietary guidelines.

**Adherence**	**Score**	**% phone calls of subject's report for full maintaining instructions to reduce AGEs in AGEs-lowering arm/for good glycemic control**
1	Very high adherence	≥80%
2	Good adherence	60–80%
3	Partial adherence	40–60%
4	Lack of adherence but intention to adhere more in the future	≤40%. Subject report on his desire to improve adherence and proceed the trial
5	Lack of adherence and no intention to adhere more	≤40%. Subject does not report on his desire to improve adherence.

### Outcome Measures

The following outcomes were assessed upon inclusion in the study (baseline), and 6 months later, immediately after participants completed the intervention (post intervention).

#### Blood Tests

Fasting blood samples were drawn and analyzed locally for glucose, insulin, HbA1C%, lipid profile, complete blood count, urea, and creatinine. In addition, blood tubes were centrifuged and serum was separated into tubes stored at −80°C for future analysis.

#### AGEs Analysis

AGEs were measured in serum samples by liquid chromatography–mass spectrometry (LC-MS) using internal stable heavy isotope substituted standards (Prevent AGE Healthcare Technology). Analysis was performed in duplicates in a blinded fashion on the serum filtrate following centrifugation through 10-K cutoff Amicon filter. This fraction contains free AGE as well as peptides of various sizes, and the analytical method measured the free products. An Agilent model 6490 Triple Quadrupole MS system with a 1290 Rapid Resolution LC system was used to detect analytes. All AGEs were separated and analyzed using Waters X-select HSS T3 column (2.5 mm, 2.1 3 150 mm) with a mobile phase gradient of methanol and water with 0.20% heptafluorobutyric acid. Total analysis time ran 19 min. Five dicarbonyl-derived AGEs were assessed: Ne-carboxymethyl lysine (CML), Ne-carboxyethyl lysine (CEL), glyoxalhydroimidazolone (G-H1), methylglyoxal hydroimidazolone (MG-H1), and 3-deoxyglucosone hydroimidazolone (3DG-H1).

#### Nutritional Assessment

Nutritional assessment included Food Frequency Questionnaires (FFQ) and AGEs questionnaires (described above in the *Face-to-face screening* section). The FFQ questionnaire was specifically designed to assess nutrition in the elderly and is commonly used in this population (41). The questionnaire includes 126 items and was administrated by the study dietitian.

#### Comprehensive Neuropsychological Assessment

A neuropsychological assessment was performed by an experienced neuropsychologist specializing in the cognitive assessment of older adults. A paper-and-pencil battery, administered in face-to-face sessions, included commonly used cognitive tests presenting a range of cognitive domains including attention, executive functions, language, and memory (Supplement B). Neuropsychological test scores were transformed into *Z* scores using the baseline mean and standard deviation and averaged for each domain (42). An overall cognition measure averaged the scores of all four domains. We also invited participants to repeat cognitive testing at 12, 6 months after the intervention.

#### Neuroimaging

Scans were acquired on a 3-T Philips Ingenia scanner using a 32-channel head radio frequency (RF) coil. Participants underwent brain MRI for cerebral blood flow (CBF) quantification using an Artierial Spin Labeling (ASL) sequence (43). Further details on the MRI acquisition can be found in Supplement C.

#### Anthropometric Measures and Clinical Scales

Weight, height, and waist circumference were measured by the study dietitian using the same equipment, and BMI was calculated [height (m)^2^/weight (kg)]. Participants wore their shoes and clothing to avoid discomfort. Waist circumference was measured in centimeters at the level of the umbilicus. Smoking habits, demographics, physical activity (Minnesota Leisure Time Physical Activity Questionnaire) (44), independence (IADL questionnaire) (45), and activities of daily living (ADL questionnaire) (46) were assessed.

#### Blinding

Although allocation to the diet intervention was randomly assigned, the participant and study dietitian could not be blinded to diet. All other staff members involved in measurement of any outcome were blind to the assignments, including the neuropsychologist, the MRI technician, and the lab technician where the blood tests were analyzed. Data entry was done by a research assistant who was also blinded to group assignments.

### Sample Size Calculation

In keeping with the pilot nature of this study, and according to CONSORT guidelines for pilot and feasibility studies, we did not calculate a formal sample size (33). Currently, there are no published data on AGEs-reduction interventions on cognition in older adults. Various authors have discussed the minimal sample size required for pilot studies (33). Here, we followed Whitehead et al. (47) who recommend at least 25 people per arm in the pilot study if the intention is to power a future trial with 90% power where the expected effect size is small. A recent meta-analysis of nutritional interventions for cognition found that the overall effect size (measured by standardized mean differences) for verbal and visual memory domains was small (=0.19) (48). With respect to attrition, a recent trial of AGEs reduction of participants with metabolic syndrome found 20 and 35% attrition rate in the regular AGEs arm and low-AGEs arm, respectively (19). Thus, for a 30% average attrition, we targeted 72 participants at baseline. Our aim was to provide initial estimation of the effect size to allow robust power calculations for a future definitive trial.

### Statistical Analysis

Alpha of 0.05 was used for significance levels. SPSS version 23 (IBM Corp., Armonk, NY, USA) was used for all analyses. Descriptive statistics were used to assess recruitment and attrition rates. Baseline demographic and clinical characteristics are presented descriptively as proportions or as means with standard deviations.

#### Analytic Plan for Results of Effects of the Diet on Biology, Cognition, and CBF

The study will be analyzed by an intent-to-treat (ITT) primary analysis, with a secondary per-protocol (PP) analysis. The ITT analysis will include all randomized participants, regardless of any protocol deviation including non-adherence, adverse events, or loss of follow-up. To be considered fully compliant in the PP analysis, participants must have reported at least 80% good compliance with low AGEs cooking modifications or following standard T2D nutritional guidance for good glycemic control in weekly phone conversations.

AGEs and other biomarkers reduction in serum from baseline to 6 months will be analyzed using two-sample *t*-test or Mann–Whitney. χ^2^-test will be used for categorical outcomes.

For the exploratory aim of estimating effect size for future RCTs, global and domain-specific cognitive outcomes will each be analyzed as *z*-scores using a linear regression model with time of assessment (baseline or 6 months) as the within-participants factor, treatment group (dietary AGEs reduction vs. control) as the between-participants factor, and baseline value of the outcome measure as the covariate. CBF will be analyzed similarly with the linear regression model.

## Results

### Recruitment

**Figure 1:**
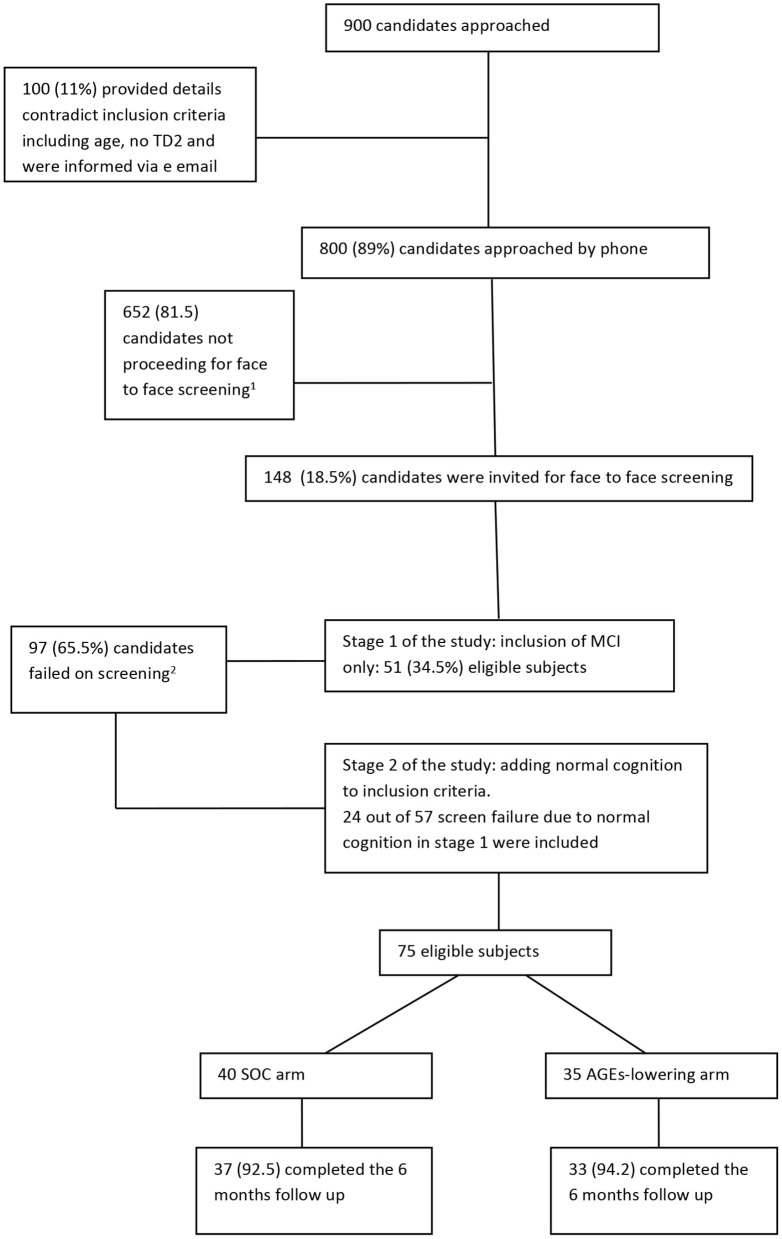
Flow chart of study recruitment and follow-up. ^1^Main reasons for not proceeding to a face to face screening were (order by prevalence): not reporting to have memory decline other than forgetting names, not having type 2 diabetes but impaired fasting glucose, no care giver available, portable issues, vegetarian, participating in other clinical trials, not interested, having other medical condition that unable them to participate such as CKD, active cancer or other neurological condition. ^2^Main reasons for screen failure (*n* = 97) were: 57 had normal cognitive status, 13 with mild depression, 9 had other health conditions that are not eligible such as brain surgery in the last year, chronic renal failure, 8 changed their mind or informant declined to cooperate, 4 had very low consumption of AGEs, 2 were advised to take medication for memory problems, 2 with CDR=1, 1 with major anxiety, 1 not fluent in Hebrew for cognitive tests.

Recruitment of the study began in July 2016 and ended in December 2018. A flow chart describing the study recruitment and follow-up is given below (Figure 1). Nine hundred candidates have shown interest to participate in the study, of whom 100 gave information that do not meet the eligibility criteria (e.g., age, impaired fasting glucose, and not T2D) and received an email informing them of their ineligibility. Out of the 800 candidates that were contacted by phone to ascertain eligibility, 148 were invited for a face-to-face screening. The most common reason for ineligibility at this stage was that the cognitive complaints were limited to a SMC (65%) that focused only on forgetting names or locating household items. Such complaints, on their own, do not indicate a clinically detectable cognitive impairment but rather normal aging. Other reasons for ineligibility at this stage are specified in Figure 1. The first stage of recruitment (i.e., recruitment of only MCI) was 27 months (between July 2016 and September 2018) and 52 participants were eligible; 23 participants were excluded due to inconsistent MOCA and CDR results impeding a clear ascertainment of cognitive status. Because it took 27 months to recruit 52 eligible participants under the original eligibility criteria (1.9 participants per month), we expanded the eligibility criteria to include cognitively normal diabetics. At the second stage of recruitment (October 2018–December 2018), we approached previously screened candidates who were originally ineligible for enrollment in the trial because their cognition was normal. Twenty-four candidates that were screened were approached in the order in which they had been screened. All of those invited (100%) agreed to participate. In total, 75 participants were randomized.

Of the different recruitment strategies, the mass mailing for people who wanted to receive information on health issues ultimately yielded the most study participants (*n* = 57). Publication of the study in websites and forums for clinical trials and diabetes yielded 11 participants, and referring participants from other observational or screen failure clinical trials yielded the rest (*n* = 7). Participants recruited through mass mailing were more educated than those recruited from other studies or websites and forums combined (14.8 ± 2.5 vs. 13.2 ± 3.1, Pv = 0.04). We did not find any differences in age or sex between the recruitment strategies. The total cost of advertising the study in social media and websites was 5,800 USD.

### Retention

Seventy participants (93%) completed 6 months of follow up. Three withdrew from the SOC arm and two withdrew from the AGEs-lowering arm. The withdrawal rate was not statistically different between the two arms (*p* = 0.757). Reasons for withdrawal from the trial in the SOC arm were deterioration in angina pectoris symptoms in one subject who could not attend the 6-month visit (the neuropsychology assessment alone was done at the participant's home); sickness of one subject's husband; and one lost to follow-up, who showed very low adherence to diet and regularly failed to answer the phone. In the AGEs-lowering arm, there were two lost to follow up; one participant showed very low adherence as reflected in the weekly phone conversation and another participant reported very high adherence during the trial but did not want to come to the follow-up visit. The average number of phone conversation made during the 6 months of follow up was 16.52. On average, a call was made every 11 days and not on a weekly basis as initially planned due to unanswered calls and holidays.

### Adherence With the Dietary Guidance

Adherence to T2D dietary guidelines was similar with very high adherence, good adherence, and partial adherence, respectively, for the two arms (*p* = 0.962): 43.2, 45.9, and 10.8% for the AGEs-lowering arm and 42.9, 47.1, and 10% for the SOC arm. Of the 33 participants who completed the study in the AGEs-lowering arm, 19 (57.5%) were scored with very high adherence, 10 (30.3%) with good adherence, and 4 (12.2%) with partial adherence for the low-AGEs adherence scale. Cognition status (MCI vs. normal cognition) was not associated with adherence to T2D (Pv = 0.338) or low-AGEs dietary guidelines (Pv = 0.352). None of the participants who finished the 6 months follow-up scored 4/5, representing no adherence.

### Participants' Characteristics

Participants' baseline characteristics are provided in Table 3. The mean age was 71.6 ± 4.07; 74.7% were males. Similarly to other elderly participants in prospective cohorts (49), our sample represented educated and motivated participants with an average of 14.4 ± 2.74 years of education and an average HbA1C% of 6.67 ± 0.67, reflecting good glycemic control. Participants' consumption of macronutrients show that carbohydrates account for <50% of the daily consumption. Participants reported AGEs consumption with a mean of 22.65 ± 6.47 kU/day. There were no significant differences between the groups at baseline on any of the variables, indicating that randomization was successful.

**Table 3:** Baseline characteristics (*n* = 75).

**Variable**	**AGEs-lowering diet (*n* = 35)**	**Control diet (*n* = 40)**	**Pv**
Age (years)	71.91 ± 4.20	71.42 ± 3.99	0.57
**Gender**, ***n*** **(%)**			
Female	8 (22.9)	11 (27.5)	0.64
Male	7 (77.1)	29 (72.5)	
Education (years)	13.89 ± 2.88	14.93 ± 2.62	0.10
**Family history of dementia**			
*N* (%)			0.61
No family history	23 (65.7)	24(60)	
**Smoking**, ***n*** **(%)**			
Never	19 (54.3)	15 (37.5)	
In the past	14 (40)	2 (5.7)	0.31
Current	23 (57.5)	2 (5)	
**Medication for T2D**, ***n*** **(%)**			
All but insulin	29 (82.9)	29 (72.5)	
Insulin included	6 (17.1)	9 (22.5)	0.32
Diet only	0	2 (5)	
MoCA	23.14 ± 2.18	23.25 ± 2.23	0.80
GDS	2.37 ± 1.71	2.53 ± 1.66	0.69
ADL	99.43 ± 2.35	99.75 ± 1.10	0.84
Physical activity (METS/day)	148.75 ± 130.92	118.63 ± 115.61	0.22
Waist circumference (cm)	106.74 ± 10.77	106.05 ± 10.81	0.78
BMI (kg/m^2^)	30.02 ± 4.28	29.55 ± 3.58	0.75
CRP (mg/L)	4.79 ± 5.28	3.48 ± 4.22	0.10
Insulin (mU/L) (*n* = 57)^a^	15.24 ± 10.97	15.11 ± 9.41	0.64
HbA1C %	6.63 ± 0.72	6.71 ± 0.67	0.59
HOMA-IR^a^^,^^b^ (*n* = 57)	5.04 ± 4.12	4.53 ± 2.35	0.81
Creatinine clearance^c^ (ml/min)	84.75 ± 26.27	85.86 ± 22.35	0.84
Estimated AGEs intake (KU/day)	23.25 ± 6.48	22.14 ± 6.50	0.46
Protein (g)	94.89 ± 27.71	96.15 ± 27.44	0.73
Fat (g)	83.21 ± 25.08	82.70 ± 30.78	0.62
Carbohydrates(g)	232.94 ± 66.67	241.75 ± 60.45	0.53
Energy (kcal)	2012.26 ± 488.83	2059.03 ± 553.68	0.86

*All values expressed as mean ± SD*.

*MoCA, Montreal cognitive assessment; GDS, Geriatric depression scale; ADL, Activity of daily living; BMI, Body mass index; CRP, C-reactive protein; HbA1C, glycated hemoglobin; HOMA-IR, Homeostatic model assessment of insulin resistance*.

a *Insulin mean and HOMA-IR include only participants who do not take insulin*.

b HOMA-IR was calculated as insulin (mu/L)^*^glucose (mg/dl)/405.

c *Creatinine clearance was calculated by the Cockcroft and Gault equation*.

## Discussion

The current paper describes the methodology, recruitment, adherence measurements, and the sample baseline characteristics of a pilot study assessing dietary AGEs reduction in older adults with T2D. Our study population is different from that of previous trials in two aspects. First, we have tested older adults (mean age, 71.6) with T2D while the age of the participants in previous trials of AGEs reduction in diabetics ranged 60–62 (18, 50). As age is positively correlated with circulating AGEs (11) and negatively with kidney function (51), a decade difference may be significant for the AGEs' pool due to both higher accumulation and decreased disposal. As such, we hypothesized that older adults with T2D may respond differently to a low-AGEs diet. Second, our trial is the first to recruit participants with cognitive impairment for intervention to reduce AGEs. The results showed that adherence to the dietary guidelines was similar in participants with impaired and normal cognition, supporting the feasibility of the program in this population. In terms of feasibility of the dietary intervention *per se*, the high adherence to dietary AGEs reduction among 57.5% of participants suggests that older adults with T2D population is able and willing to commit to a low-AGEs diet that is closely supervised by a nutritionist. We focused the study on older adults with T2D as we anticipated this as an optimal population to target for AGEs modification to benefit cognition, since T2D individuals have greater accumulation of AGEs due to hyperglycemia (12) and are also at higher risk for cognitive decline and dementia (9). Our original intention was to recruit only participants with MCI. Under the eligibility criteria in the first stage of recruitment, the overall screen failure rate for 900 participants screened was 17 for every eligible subject, emphasizing the great challenge in recruiting MCI with T2D. The main reason for ineligibility through telephone screening was that what candidates reported as memory deficit was actually normative. In the face-to-face screening, 65% did not meet the inclusion criteria and 58% of those screen failures were due to normal cognition. Low rates of recruitments of MCI participants from both community (52) and clinic settings (53) and the low sensitivity of SMCs alone to find eligible participants have been shown in prior studies. A community-based trial found that only 44% of those who applied with memory concerns following newspaper advertisement had MCI while 47% were classified with normal cognition in the tests with an average MOCA of 27 (52). Kirsebom et al. showed that cognitive performance of participants referred by memory clinics compared to self-referral is significantly lower in MCI (54). In other words, previous studies support our finding that SMCs from the general population do not distinguish participants with MCI and that involving several memory clinics may be a more successful strategy for identifying a large number of eligible participants with MCI. Since several studies indicated that use of a mutual informant with self-report of cognitive decline may better represent cognitive changes, our screening process also included an informant (51, 52).

We found that the most productive recruitment tool, which was the source of 76% of our participants, was mass mailing to a list of 30,000 people who indicated they wished to receive email information about health. We sent this email eight times at 3-month intervals, allowing enough time for updating the list. We also advertised in relevant internet forums and websites and used referrals from other studies as previously suggested (55), but those yielded a relatively small number of eligible participants. We found that although potential participants who approached us from different sources did not differ in age or sex, participants from mass mailing for people interested in receiving information about health had higher education and thus may not have been representative of the general older adult population. Thus, it is likely that they represented motivated participants wishing to improve their health and therefore more willing to volunteer for such trials.

Our findings demonstrate that it is possible to achieve high compliance with our study protocol as shown by the excellent telephone contact (mean of 16.52) and low withdrawal rate (6%). Other AGEs-lowering clinical trials (19) experienced a much higher withdrawal rate (27%) over the course of 1 year. Similarly, another behavioral intervention study in elderly with MCI that included cognitive training, yoga support groups, and wellness education reported a 17% dropout at 1 year (53). Our low attrition rate may be due to the intense follow up during the 6-month study, which helped participants maintain the guidelines and to ask questions about their interpretation of the guidelines (e.g., “can I use the oven if I cover the food?” “what food should I choose to eat if I have an event next week,” etc.). Along with a high retention rate, participants' adherence with the instructions was high as well; 57.5% of participants from the AGEs-lowering arm reported that they followed the guidelines to reduce AGEs in at least 80% of the phone conversations. Another 30.3% reported that they maintained the guidelines 60–80% of the time and only 12.2% reported maintaining the guidelines <60% of the time. Nevertheless, measuring adherence to dietary guidelines is a challenge, and we cannot rule out self-reporting bias. Questionnaires for diet adherence such as the MEDI score questionnaire (56) or self-reported scale of adherence with T2D recommendations (57) usually assess the intake of certain food items. Our study did not ask the participants to score their own adherence, as self-reported adherence may be biased. Instead, we used an adherence score built on the dietitian perspective based on numerous phone conversations, suggesting that the adherence measure is less vulnerable to information bias. To our knowledge, this study is the first to use a dietitian's perspective scale for adherence measurement. In future studies, both self-reported and dietician-based adherence assessments should be collected and their correlations should be examined to validate the advantages of one over the other as adherence scales may indicate in real time who needs a higher rate of monitoring in order to increase adherence. The high adherence to the SOC guidelines for T2D in both arms might have contributed to a partial reduction in serum AGEs, which was observed also in the “active” SOC control arm. This may have limited our ability to assess the true effect of decreasing circulating AGEs compared to no change or increase over time that would be expected in a passive control. We have recently showed that that the reduction of serum AGEs in the intervention arm was not attributed to any metabolic benefits other than reduced AGEs intake (32). However, a future RCT may require a passive intervention arm to definitively determine the benefit of the intervention. While this may pose ethical challenges regarding equipoise, it may be acceptable to randomize participants to a waiting list, to receive SOC guidance at baseline without any further involvement during the follow up, or to use a crossover design with passive and active interventions in random order.

While our study highlights the feasibility of AGEs-lowering diet for cognitive protection in older diabetics, it also has several limitations. An unavoidable limitation of the design (overt dietary interventions) is that neither the investigators nor the patients were blind to the intervention. We attempted to minimize this effect by providing both arms tailored from dietary recommendations and followed both groups equally. In addition, the average HbA1C of 6.6% ± 0.6% suggests that our participants maintain good health in general so that their adherence to such a diet may not be generalized to the population of older adults with T2D at large. In addition, the successful recruitment through mass mailing of people wishing to learn about health issues also raise the potential of selection bias. A sample including more severe diabetics or non-diabetics with other metabolic conditions that do not follow regularly dietary guidelines may benefit more from such intervention and should be investigated in the future.

In conclusion, we described the methods, recruitment, and adherence of a pilot study of low-AGEs diet in elderly with T2D and brain-related outcomes. The pilot study emphasizes the challenge in recruiting MCI participants from the general population and shows that mass mailing and social media advertising are a good strategy for recruiting candidates. We found that including an informant and an objective global cognition tool (MoCA) rather than relying on subjective complaints alone was important for MCI screening. Lastly, our findings suggest that an intensive follow-up during the intervention might help reduce attrition in a study aimed at promoting health-related behavioral change.

## Data Availability Statement

The raw data supporting the conclusions of this article will be made available by the authors, without undue reservation.

## Ethics Statement

The studies involving human participants were reviewed and approved by Sheba medical center approval number 2206-15-SMC. The patients/participants provided their written informed consent to participate in this study.

## Author Contributions

RL conducted the trial, analyzed the study data, and wrote the initial draft. IG and SS were the trial physicians and signed up the participants on informed consent. AL managed the neuro-imaging section of the trial and participated in writing of the manuscript. JU and WC served as scientific advisors and participated in writing or technical editing of the manuscript. PB was responsible for the analysis of advanced glycation end products. MZ coordinated the trial and was responsible for the data entry. MS and AT initiated the trial plan, analyzed data, and revised and finalized the manuscript. All authors contributed to the article and approved the submitted version.

## Conflict of Interest

The authors declare that the research was conducted in the absence of any commercial or financial relationships that could be construed as a potential conflict of interest.
