# Supplementary material for: Design and Feasibility of a Randomized Controlled Pilot Trial to Reduce Exposure and Cognitive Risk Associated With Advanced Glycation End Products in Older Adults With Type 2 Diabetes
Source: Front Nutr. 2021 Feb 15;8:614149. doi: 10.3389/fnut.2021.614149 (PMC7917071; doi:10.3389/fnut.2021.614149)
Supplement: Supplementary file 1 [file Table_1.DOCX]

| **Supplement document for manuscript**   1. **AGEs questionnaire** | | | | | | | | | | | | | | | | | |
| --- | --- | --- | --- | --- | --- | --- | --- | --- | --- | --- | --- | --- | --- | --- | --- | --- | --- |
| **Food Category** | **Weekly Consumption** | | | | | | **Serving Size** | | | | **Preparation Method** | | | | | **AGE SCORE** | |
|  | **0** | **1** | **2, 3** | **4-6** | **Daily** | **> 1/day** | **<3 oz** | **3-6 oz** | | **> 6 oz** | **Boil/Can Stew** | **Pan Fry** | **Roast** | **Broil Grill** | **Oven fry Deep Fry** | **Item** | **Group** |
| **Beef** | **0** | **1** | **3** | **5** | **7** | **8** | **1** | **2** | | **3** | **3** | **3** | **6** | **7** |  |  |  |
| Burgers, pan fried, not fast food |  |  |  |  |  |  |  |  | |  |  |  |  |  |  |  |  |
| Chopped/Ground- Meatballs, Meatloaf |  |  |  |  |  |  |  |  | |  | **all preps** |  |  |  |  |  |  |
| Cubes or Strips |  |  |  |  |  |  |  |  | |  |  |  |  |  |  |  |  |
| Roasts |  |  |  |  |  |  |  |  | |  |  |  | **X** |  |  |  |  |
| Steaks, Ribs |  |  |  |  |  |  |  |  | |  |  |  |  |  |  |  |  |
| **Poultry** | **0** | **1** | **3** | **5** | **7** | **8** | **1** | **2** | | **3** | **2** | **5** | **5** | **6** | **10** |  |  |
| Ground-Meatballs, Meatloaf |  |  |  |  |  |  |  |  | |  |  |  |  |  |  |  |  |
| Chicken |  |  |  |  |  |  |  |  | |  |  |  |  |  |  |  |  |
| Turkey |  |  |  |  |  |  |  |  | |  |  |  |  |  |  |  |  |
| Chicken Nugg, **5 pc** |  |  |  |  |  |  |  |  | |  |  |  |  |  | **X(9)** |  |  |
| **Ham & Pork** | **0** | **1** | **3** | **5** | **7** | **8** | **1** | **2** | | **3** |  |  | **2** | ***4*** |  |  |  |
| Deli Ham |  |  |  |  |  |  |  |  | |  |  |  | **X** |  |  |  |  |
| Pork Chop,fried, broil |  |  |  |  |  |  |  |  | |  |  |  |  | **X** |  |  |  |
| **Fish** | **0** | **1** | **3** | **5** | **7** | **8** | **1** | **2** | | **3** | **Smoked Can-W** | **Boiled Can-O** | **Poach Rst/Br** | **Sal/Tun Broil** | **Oven Fry Deep Fry** |  |  |
|  |  |  |  |  |  |  |  |  | |  |  |  |  |  |  |  |  |
|  |  |  |  |  |  |  |  |  | |  | **0.5** | **1** | **2** | **5** | **8** |  |  |
| Salmon |  |  |  |  |  |  |  |  | |  |  |  |  |  |  |  |  |
| Tuna |  |  |  |  |  |  |  |  | |  |  |  |  |  |  |  |  |
| Whiting, Trout, Other |  |  |  |  |  |  |  |  | |  |  |  |  |  |  |  |  |
| **Food category** | **Weekly consumption** | | | | | |  |  | |  |  |  |  |  |  |  |  |
|  | **0** | **1** | **2,3** | **4-6** | **Daily** | **> 1/day** | **<3 oz** | **3-6 oz** | **> 6 oz** | |  |  |  |  |  |  |  |
| **Tofu** | **0** | **1** | **3** | **5** | **7** | **8** | **1** | **2** | | **3** |  | **Raw** | **Fry/Br** |  |  |  |  |
|  |  |  |  |  |  |  |  |  | |  |  | **1** | **4** |  |  |  |  |
|  |  |  |  |  |  |  |  |  | |  |  |  |  |  |  |  |  |
| **Processed meats** | **0** | 1 | 3 | 5 | 7 | 8 | **<1sv** | **1-2 sv** | | **>sv** |  | **Boil/can** | **Broil** | **Pan Fry** |  |  |  |
|  |  |  |  |  |  |  | **1** | **2** | | **3** |  | **1** | **3** | **3** | **5.5** |  |  |
| Bacon, **1 sl** |  |  |  |  |  |  |  |  | |  |  |  |  |  | **X** |  |  |
| Sausage, **2 sm links** |  |  |  |  |  |  |  |  | |  |  |  |  | **X** |  |  |  |
| **Eggs** | **0** | **1** | **3** | **5** | **7** | **8** | **1** | **2** | | **3** |  | **0.5** |  | **1** |  |  |  |
| Cooked WholeEgg,**1** |  |  |  |  |  |  |  |  | |  |  | **X** |  |  |  |  |  |
| Fried Whole Egg, **1** |  |  |  |  |  |  |  |  | |  |  |  |  | **X** |  |  |  |

| **Food Category** | **Weekly Consumption** | | | | | | **Serving Size** | | | **Preparation Method** | | | | |  |  |
| --- | --- | --- | --- | --- | --- | --- | --- | --- | --- | --- | --- | --- | --- | --- | --- | --- |
|  | **0** | **1** | **2, 3** | **4-6** | **daily** | **>1/day** | **< 1 sv** | **1-2 sv** | **≥ 3 sv** | **Fat Free** | **Low Fat** | **Regular** | | | **Item** | **Group** |
| **Cheese** | **0** | **1** | **3** | **5** | **7** | **8** | **1** | **2** | **3** | **0** | **1** | **2** | **3** | **4** | **AGE Score** | |
| American Cheese,**slice** |  |  |  |  |  |  |  |  |  |  |  |  | **X** |  |  |  |
| Low Fat Amer Cheese, **1 sl** Mozzarella Cheese, **1 oz** |  |  |  |  |  |  |  |  |  |  | **X** |  |  |  |  |  |
| Parmesan,**tbsp**, Feta, **1 oz** |  |  |  |  |  |  |  |  |  |  |  |  | **X** |  |  |  |
| Brie, Swiss, **1 oz** Cottage Cheese, **1/2 cup** |  |  |  |  |  |  |  |  |  |  |  | **X** |  |  |  |  |
| Baked Mac & Cheese, **.5C** |  |  |  |  |  |  |  |  |  |  |  |  |  | **X** |  |  |
| Cooked Mac & Cheese,**.5C** |  |  |  |  |  |  |  |  |  |  |  |  | **X** |  |  |  |
| **Fats** (added to foods) | **0** | **1** | **3** | **5** | **7** | **8** | **1** | **2** | **3** |  | **Low Fat** | **Regular** | | |  |  |
|  |  |  |  |  |  |  |  |  |  | **0** | **1** | **1.5** | **2** | **2.5** |  |  |
| Butter/Margarine, **1 tsp** |  |  |  |  |  |  |  |  |  |  |  | **X** |  |  |  |  |
| Soft Margarine, **1 tsp** |  |  |  |  |  |  |  |  |  |  | **X** |  |  |  |  |  |
| Cream Cheese, **1 tbsp** |  |  |  |  |  |  |  |  |  |  |  | **X** |  |  |  |  |
| Peanut Butter, **2 tbsp** |  |  |  |  |  |  |  |  |  |  |  |  | **X** |  |  |  |
| Cooking Oil, **1 tbsp** |  |  |  |  |  |  |  |  |  |  |  | **X** |  |  |  |  |
| Mayonnaise, **1 tbsp** |  |  |  |  |  |  |  |  |  |  |  | **X** |  |  |  |  |
| Nuts, **1 oz** |  |  |  |  |  |  |  |  |  |  |  |  |  | **X** |  |  |
| **Convenience Foods** | **0** | **1** | **3** | **5** | **7** | **8** | **1** | **2** | **3** | **0.5** | **1** | **1.5** | **2** | **3** |  |  |
| Frozen Waffle, toasted **1** |  |  |  |  |  |  |  |  |  |  | **X** |  |  |  |  |  |
| Potato Chips(not corn),**1oz** Granola Bar, hard **1** |  |  |  |  |  |  |  |  |  |  | **X** |  |  |  |  |  |
| Cookies, Crackers,Pretzels Melba Toast, **1 oz** |  |  |  |  |  |  |  |  |  | **X** |  |  |  |  |  |  |
| Biscotti, Croissant, **1 oz** |  |  |  |  |  |  |  |  |  |  | **X** |  |  |  |  |  |
| Fast Food French Fries |  |  |  |  |  |  | **sm** | **med** | **lg** |  | **X** |  |  |  |  |  |
|  |  |  |  |  |  |  |  |  |  |  |  |  |  |  |  |  |
| **Food Category** | **Weekly consumption** | | | | | | **Serving size** | | | **Food preparation** | | | | | |  |
| **Misc,not reported above** | **0** | **1** | **2-3** | **4-6** | **daily** | **>1/day** | **<1 sv** | **2-3 sv** | **≥ 3 sv** |  |  |  |  |  |  |  |
|  | **0** | **1** | **3** | **5** | **7** | **8** | **1** | **2** | **3** | **4** | **5** | **6** | **8** | **11** |  |  |
| FF Cheeseburger, **1/4 lb** |  |  |  |  |  |  |  |  |  |  |  |  | **X** |  |  |  |
| 6 in Hero-Turkey & Mayo |  |  |  |  |  |  |  |  |  |  |  | **X** |  |  |  |  |
| 6 in Hero-Ham, Ch, Mayo |  |  |  |  |  |  |  |  |  |  |  | **X** |  |  |  |  |
| Grilled Cheese, **2 sl ch** |  |  |  |  |  |  |  |  |  |  |  | **X** |  |  |  |  |
| Pizza, **1 large slice** |  |  |  |  |  |  |  |  |  |  |  |  |  | **X** |  |  |
| Frankfurter, broiled, **1** |  |  |  |  |  |  |  |  |  |  |  |  | **X** |  |  |  |
| Frankfurter, boiled, **1** |  |  |  |  |  |  |  |  |  |  | **X** |  |  |  |  |  |
| Lunch Meat (Salami, Bologna), **3 oz** |  |  |  |  |  |  |  |  |  | **X** |  |  |  |  |  |  |
|  |  |  |  |  |  |  |  |  |  |  |  |  |  |  | **Total** |  |

AGE Quick Score

Script and Protocol For Administering Questionnaire

*Please complete the identifying information at the top of the questionnaire prior to meeting with the subject.*

*Please read out loud to subject:*

Today we are interested in finding out about some of the foods you may have eaten in the past week. I will be asking about foods you may have eaten for meals and/or snacks. You may have eaten some of the foods, all of the foods, or none of the foods. You also may have eaten many other foods that I will not be asking about today.

There are no right or wrong answers.

It will take us about 15 minutes to complete this questionnaire.

I will ask about a number of foods. If you have not eaten a particular food I will merely move on to the next food. If you have eaten the food I have named, I will ask you how many times you ate it during the past week. I will also ask you about the portion or amount you ate and then I will ask you about how the food was cooked or prepared.

I will show you a guide to help you estimate the portions you are eating using your hands.

For example, 3 ounces of meat is about the size of the palm of your hand. Some people eat portions that are the same as the guide; some people eat portions that are smaller than those shown; still, others will eat portions that are much larger than the guide.

Do you have any questions?

*Answer the subject’s questions.* *Repeat instructions if necessary. (When all questions have been answered and instructions have been clarified, continue.)*

Then I will begin.

*Record the subject’s answers to the questions. Do not comment on the foods he or she consumes or does not consume.*

*Please read the foods in the order in which they appear on the questionnaire. Begin with Beef. If the subject says “no” put a slash through the entire category under the “never” column and proceed to Poultry. If the subject says “yes,” ask about burgers, pan-fried, not fast food. If the answer is yes, then ask how many times in the past week the subject ate the food. Mark the appropriate box. Next ask about the serving size the subject may have consumed. Point to the portion guide sheet and ask the subject to compare his or her portion to the picture of a similar food. For example, was the burger the size of the palm of the subject’s hand, half a palm, or more than one palm. If several, how many. Mark the appropriate box. Ask about each food in the category in the same fashion before moving to the next food category.*

*HINT: Use a ruler to highlight one line at a time. This will help you record all the answers for each food on the same line.*

*Continue through Cheese.*

Now I am going to ask about Fats you may have used in cooking, added to your food, or spread on bread and/or crackers. In the past week have you eaten butter or margarine? How many times? If “yes” how much at one time? …

Now I will ask about some other items. We are almost finished*.*

*Ask about the remaining two categories. Complete the questionnaire. Be sure all questions have been answered.*

Now we have completed the questionnaire. Thank you for sharing this important information with us today. Everything you have shared will remain completely confidential.

*Carefully tally up the scores for each item. Place a” zero” in the scoring column for any category which the subject excludes completely. Please write clearly. You may use a handheld calculator to assist in this task. If you discover that you have made an error, draw a line through the incorrect answer. Write your initials and today’s date (e.g. sg 4-13-04) next to the error. Do not use white out to make corrections. Total up all the items from page 1 and page 2 to determine the AGE score for the week. Divide by 7 to obtain an average daily score. Thanks for your help!!!*

**B: Cognitive assessments**

| **Table 1- cognitive assessments** | | |
| --- | --- | --- |
| Domain | Test | Information |
| Memory | Logical Memory (Story A) immediate and delayed | A short story is read to the participant, who recalls the story immediately after it is read (immediate recall) and after 20 minute delay (delayed recall). The possible scores for immediate recall and for delayed recall range from 0-25 |
|  | ADAS-COG Word List immediate memory | Subjects read aloud 10 unrelated words on printed cards, and are asked to immediately recall as many as possible, in three learning trials. |
|  | ADAS-COG Word List delayed memory | The participants try to recall the 10 words from the Word List Memory task after a 15-minute delay. During this delay, other tests were conducted. Scores range from 0-10. |
|  | Word List Recognition | Participant try to recognize12 words presented earlier, when presented also with 12 distractor words. There are 12 correct responses for the target words and 12 for the distractor words. Scores range from 0-24. |
| Executive function | Constructional Praxis (Praxis) | Participants are required to copy four line drawings presented in order of increasing complexity (circle, diamond, overlapping rectangles, and cube). Scores range from 0-11. |
|  | Trail Making Test (A&B) | In Part A (Trails A), numbers are randomly ordered in a page, which the participant connects in ascending order by drawing a line from number to number. Trails A relies heavily on motor speed. In Part B (Trails B), numbers and letters are randomly arranged in a page, and the participant connects them by drawing a line while alternating between a number and a letter in ascending order. In addition to motor speed, the switching component of Trails B requires mental flexibility. Although, the number of errors was recorded, only the time in seconds to complete each task was used in these analyses. The maximum time to complete each task was 300 seconds. |
| Attention | Digit Span (forward and backward) | An oral repetition of number sequences. For digit forward , the participant repeats the sequence verbatim. For digit backward, the repetition is in reverse. For each task, the scores range from 0-30. |
|  | Shape Cancellation (Shape) | A page with target (diamonds) and distractor shapes is given to the participant The participant crosses out only the target shapes as quickly and accurately as possible until the task is completed or four minutes have elapsed. Although, the number of errors was recorded, only time in seconds was used in these analyses. The maximum time was 240 seconds. |
| Language | Similarities | The participant states the similarities or superordinate categories of paired words (concepts or objects). Score range from 0 – 33 points. |
|  | Semantic verbal Fluency | Participants provide as many examples of the category “animal” and “fruit and vegetables” as possible in 60 seconds. |
|  | Phonemic verbal Fluency | This test requires the ability to generate as many words as possible that begin with the letters F, A, and S (phonemic fluency) in one minute for each letter. For this task, the Hebrew letters bet, gimel, and shin were used since they are widely administered in neuropsychological testing in Israel |
|  | Boston Naming Test- short version | Participant should identify the names of 15 line drawings. Scores range from 0-15. |

**C: MRI imaging**

The sequence will be acquired using background-suppressed **pseudo-continuous ASL** (pcASL), with the following parameters: TR = 4500ms; TE = 13ms; FOV = 240 x 240 x 155mm; slice thickness = 5mm no gap; matrix size=128 x 128;with a post-labeling delay of 1800 ms.**T2, FLAIR** will be acquired with the following parameters: TR 9000, TE 110, TI 2500, FOV 240x 192x 153mm acquired over 354 m thick slices, no gap, with final voxel resolution 0.75mm x 0.75mm x 4mm. **3D T1-weighted imaging** will be acquired with the following parameters: inversion-recovery prepared fast spin echo, TR 7.92, TE 3.5, FOV 256x 180x 155mm, acquired over 155axial slices with isotropic 1mm voxel resolution and no gap. Participants are asked to refrain from caffeine for 3 hours and nicotine for 1.5 hours prior to MRI.

**MRI Quality Control** is done by Sheba Department of Radiology in conjunction with Philips field physicists. Trained and certified MR technologists operate the console; scans are prescribed in a standardized fashion and assessed at the scanner for acceptable quality. Scans are downloaded to the imaging lab server immediately after the session and the post-processing procedures are implemented including an additional quality check by the study team.

**MR Image Processing:**

The CBF images will be processed using SPM12 (www.fil.ion.ucl.ac.uk/spm). The procedure involves: 1) Segmenting the high resolution T1-weighted images; 2) Co-registering the grey-matter probability maps with the CBF images. 3) Spatially normalizing the T1 volume and associated CBF image to the Montreal Neurological Institute (MNI) template, with re sampling to a 2 × 2 × 2 mm voxel size. 4) Smoothing the normalized CBF maps using an 8-mm full-width at half-maximum Gaussian kernel. We will then corrected for inter-individual variations in global perfusion by scaling each voxel in the CBF map by the mean whole-brain CBF.
